# Exploring the impact of lipid droplets on the evolution and progress of hepatocarcinoma

**DOI:** 10.3389/fcell.2024.1404006

**Published:** 2024-05-16

**Authors:** Samantha Maurotti, Nadia Geirola, Miriam Frosina, Angela Mirarchi, Francesca Scionti, Rosario Mare, Tiziana Montalcini, Arturo Pujia, Luca Tirinato

**Affiliations:** ^1^ Department of Clinical and Experimental Medicine, University “Magna Græcia” of Catanzaro, Catanzaro, Italy; ^2^ Department of Medical and Surgical Sciences, University “Magna Græcia” of Catanzaro, Catanzaro, Italy

**Keywords:** lipid droplets, liver, MAFLD, MASH, cirrhosis, hepatocarcinoma, PLINs

## Abstract

Over the past 10 years, the biological role of lipid droplets (LDs) has gained significant attention in the context of both physiological and pathological conditions. Considerable progress has been made in elucidating key aspects of these organelles, yet much remains to be accomplished to fully comprehend the myriad functions they serve in the progression of hepatic tumors. Our current perception is that LDs are complex and active structures managed by a distinct set of cellular processes. This understanding represents a significant paradigm shift from earlier perspectives. In this review, we aim to recapitulate the function of LDs within the liver, highlighting their pivotal role in the pathogenesis of metabolic dysfunction-associated steatotic liver disease (MASLD) (Hsu and Loomba, 2024) and their contribution to the progression towards more advanced pathological stages up to hepatocellular carcinoma (HC) (Farese and Walther, 2009). We are aware of the molecular complexity and changes occurring in the neoplastic evolution of the liver. Our attempt, however, is to summarize the most important and recent roles of LDs across both healthy and all pathological liver states, up to hepatocarcinoma. For more detailed insights, we direct readers to some of the many excellent reviews already available in the literature (Gluchowski et al., 2017; Hu et al., 2020; Seebacher et al., 2020; Paul et al., 2022).

## 1 Lipid droplet’ biology

Initially revealed and described by Richard Altmann and E. B. Wilson in the late 19th century (Altmann, 1894; Stern, 1967), LDs were merely considered cellular fat repositories. For decades, their biological potential remained underexplored due to the assumption that they were passive storage sites. The story shifted in 1991 when Dean Londos’ research identified specific proteins within LDs, called perilipins (PLINs), underscoring its pivotal role in lipid metabolism and LD biology. Consequently, this spurred a transformative perception on the biological significance of LDs, a view further solidified in 2009 when Tobias Walter and Robert Farese published a milestone paper highlighting the role and importance of these organelles in mammalian cells (Farese and Walther, 2009).

LDs are specialized, dynamic organelles encapsulating a hydrophobic core of stored lipids, mainly consisting of triacylglycerides (TGs) and cholesterol esters (CEs). This core is stabilized by a unique monolayer of phospholipids (Tirinato and Pagliari and Limongi and Marini and Falqui and Seco and Candeloro and Liberale and Di Fabrizio, Stem cells international, 2017, 2017). Embedded within this monolayer there are various proteins that confer functionality and regulation. On the LD surface, PLINs are predominant, serving as guardians and organizers of lipid storage and mobilization. Enzymatically active proteins, such as lipases, orchestrate the breakdown of stored lipids. Additionally, lipid biosynthetic enzymes are present, contributing to lipid homeostasis. Diverse proteins involved in membrane trafficking, signaling, and structural maintenance are also integral to LD biology, reflecting the organelle’s complex interplay with cellular processes (Roberts and Olzmann, 2020).

LD formation begins with the accumulation of neutral lipids (TGs and CEs) between the leaflets of the endoplasmic reticulum (ER) membrane. This nascent lipid accumulation leads to the budding of a lipid lens within the ER bilayer. As the lens grows, it protrudes into the cytosol, eventually pinching off to form a distinct cytosolic LD. The droplet remains bounded by a phospholipid monolayer that is derived from the ER membrane and is decorated with specific proteins that target the droplet during or post-formation (Zadoorian et al., 2023). This biogenesis process is tightly regulated by cellular energy status and involves a complex interplay of lipid biosynthetic and trafficking pathways.

### 1.1 Lipid droplets in healthy liver

**FIGURE 1:**
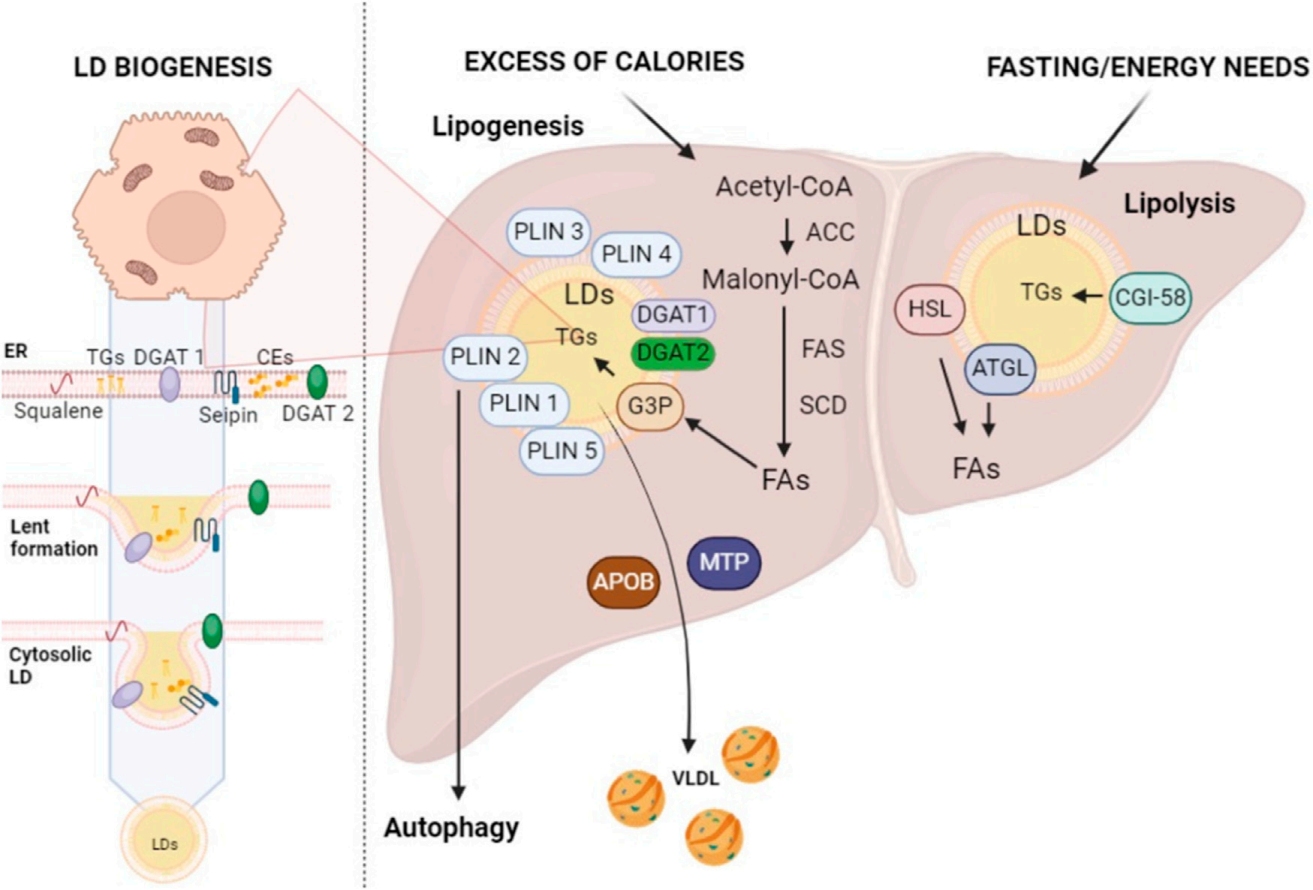
Lipid droplet biogenesis and their roles in lipogenesis and lipolysis. Enzymes found within the ER, such as DGAT1 and DGAT2, as well as squalene and seipin, play a major role in the production of LDs at the ER site. This figure schematically represents the complex metabolic processes of lipogenesis and lipolysis within the liver, highlighting the role of LDs in the storage and breakdown of fats. ACC, acetyl-CoA carboxylase; APOB, apolipoprotein B; ATGL, adipose TG lipase; CE, cholesterol esters; CGI-58, comparative gene identification 58; FAs, fatty acids; FAS, FA synthase; G3P, glycerol 3-phosphate; HSL, hormone-sensitive lipase; SCD, Stearoyl-CoA Desaturase; TGs, triglycerides; VLDL, very-low-density lipoproteins (created with BioRender.com).

LDs play an indispensable role in ensuring the healthy liver functionality, significantly contributing to various metabolic processes (Gluchowski et al., 2017). During periods of excess calories, hepatocytes store extra energy as neutral lipids within LDs through lipogenesis (Ducharme and Bickel, 2008) (Figure 1). The beginning of lipogenesis is marked by acetyl-coenzyme A (acetyl-CoA) production, which is transported into the cytoplasm for fatty acid (FA) synthesis to take place (Chandel, 2021). In this phase, acetyl-CoA carboxylase (ACC) catalyzes the acetyl-CoA carboxylation to produce malonyl-coenzyme A (malonyl-CoA), an essential component for FA synthesis (Foster, 2012; Chandel, 2021) (Figure 1). This is followed by a cascade of enzymatic reactions, led by crucial enzymes such as FA synthase (FAS), which govern the elongation of FA chains (Montesdeoca et al., 2020; Chandel, 2021) (Figure 1). These reactions systematically add carbon units, leading, with th.; e active participation of the stearoyl-CoA desaturase (SCD), to the biosynthesis of both saturated and unsaturated FAs (Dobrzyn and Ntambi, 2005). Upon synthesis, FAs can directly link to glycerol 3-phosphate (G3P) on the LD surface, initiating TG formation (Chandel, 2021). The final phase of this synthesis is facilitated by enzymes like diacylglycerol acyltransferase I and II (DGAT1 and DGAT2) (Figure 1) (Harris et al., 2011).

Once TGs have been stored within LDs, these organelles are regulated by LD-associated proteins, known as perilipins (PLINs) (Figure 1) (Bombarda-Rocha et al., 2023). These proteins play a critical role in transitioning LDs from simple structures of inactive lipid storage to fully functional organelles that are integrally involved in lipid metabolism (Bombarda-Rocha et al., 2023). There are five distinct types of perilipins (PLIN1-5), each with specific expression patterns and metabolic activations, yet all are capable of regulating lipase activity on LDs (Bombarda-Rocha et al., 2023). This diversity establishes a complex orchestrated mechanism that is directly linked to the healthy balance between lipogenesis and lipolysis (Bombarda-Rocha et al., 2023).

Specifically, PLIN2 is recognized for its role in regulating autophagy in the liver, suggesting its involvement in the dynamics of TG turnover and the management of lipid accumulations (Tsai et al., 2017).

Concerning PLINs’ specific role in lipid storage and release, it is highlighted that these proteins play a crucial role in managing enzyme access to LD surfaces, which in turn regulates TG storage and release. For instance, when phosphorylated, PLIN1 and PLIN5 can modulate lipid mobilization by enhancing lipolytic activity in response to hormonal stimuli. Similarly, the phosphorylation of PLIN2 by AMPK during activation states stimulates the lipophagy process, underscoring the complexity of the regulatory system mediated by PLINs (Bombarda-Rocha et al., 2023). TGs stored in LDs can be mobilized and incorporated into very low-density lipoprotein (VLDL) particles, synthesized in the ER, facilitating their secretion from the liver to peripheral tissues (Alves-Bezerra and Cohen, 2017). This process involves the complex interaction of lipids with proteins, particularly apolipoprotein B (apoB) and microsomal TG transfer protein (MTP). MTP is essential for transferring lipids to apoB during translation and for the formation of apoB-containing precursor particles. These precursor particles then fuse with LDs to form mature VLDL. After synthesis, these particles are organized into vesicles and sent to the Golgi apparatus. This process illustrates the complex intracellular trafficking and secretion processes essential for VLDL maturation and release. (Shelness and Sellers, 2001; Tiwari and Siddiqi, 2012; Yao et al., 2013).

On the other hand, lipolysis—a process that breaks down TGs into glycerol and FAs to provide energy - starts during fasting or other times when there is a greater need for energy (Figure 1) (Ducharme and Bickel, 2008). In this context, TGs stored in LDs undergo hydrolysis by enzymes such as adipose TG lipase (ATGL) and hormone-sensitive lipase (HSL) (Althaher, 2022). ATGL plays a pivotal role in the initial phase of TG hydrolysis on the LD surface, producing diacylglycerol (DAG) and free FAs (Figure 1) (Zimmermann et al., 2004). Subsequently, DAG is further broken down into monoacylglycerol (MAG) and FAs through the action of HSL, which is capable of hydrolyzing TGs, MAG, and cholesterol esters (CEs) (Fredrikson et al., 1981). Finally, MAG is further cleaved into glycerol and FAs by monoacylglyceride lipase (MGLs) (Zechner et al., 2012; Tardelli et al., 2020).

ATGL interacts directly with a number of proteins, which affects its lipolytic action. The primary coactivator of ATGL that facilitates the lipid core of LDs’ exposure to ATGL is recognized to be the comparative gene identification 58 (CGI-58, also known as a/b-hydrolase domain-containing 5—ABHD5) (Lass et al., 2006; Zechner et al., 2012; Tardelli et al., 2020). Since CGI-58 may regulate LD catabolism without the help of ATGL, it appears to play a multifaceted role in LD turnover, at least in the liver (Lord et al., 2016). Therefore, LDs act as a coordinating center for the harmonious regulation of lipogenesis and lipolysis (Ducharme and Bickel, 2008). The size and number of LDs can be controlled by the liver in response to a variety of stimuli, including nutritional and hormonal signals as well as metabolic demands (Crunk et al., 2013; Seebacher et al., 2020; Mashek, 2021). To coordinate the regulation of lipogenesis, pathways like the mammalian target of rapamycin (mTOR) can combine signals from growth factors and amino acids. The precise balance between lipogenesis and lipolysis is regulated by hormone signals like glucagon and insulin. The liver can therefore deal with fluctuating nutritional needs and energy levels (Crunk et al., 2013; Seebacher et al., 2020; Mashek, 2021).

The expression of key lipogenic enzymes is modulated by transcription factors called sterol regulatory element-binding proteins (SREBPs) and carbohydrate-responsive element-binding proteins (ChREBP) (Xu et al., 2013). The activity of these transcription factors is directly influenced by the cellular levels of lipids and sterols, thereby impacting lipid synthesis (Xu et al., 2013). Specifically, SREBPs enhance the expression of genes involved in lipogenesis, such as FAS and ACC, promoting the production of TGs and FAs (Mashek, 2021). Transcriptional regulation is necessary for a coordinated and efficient lipid response to cellular needs. Additionally, it is important to note that LDs in the liver engage in dynamic interactions with adipose tissue. This interaction facilitates the release of FAs through lipolysis in adipose tissue, which, upon absorption by the liver, can be assembled with TGs within LDs. This interplay underscores the interconnected nature of lipid metabolism across various tissues and highlights the liver’s role in adapting to changes in lipid availability and demand at a systemic level.

Summarizing, LDs are required in a healthy liver to store energy-producing TGs and CEs. They ensure that the liver maintains cellular energy balance by controlling lipid turnover and metabolism.

## 2 Lipid droplets dysregulation: from steatosis to hepatocarcinoma

### 2.1 LDs in metabolic dysfunction-associated steatotic liver disease (MASLD)

**FIGURE 2:**
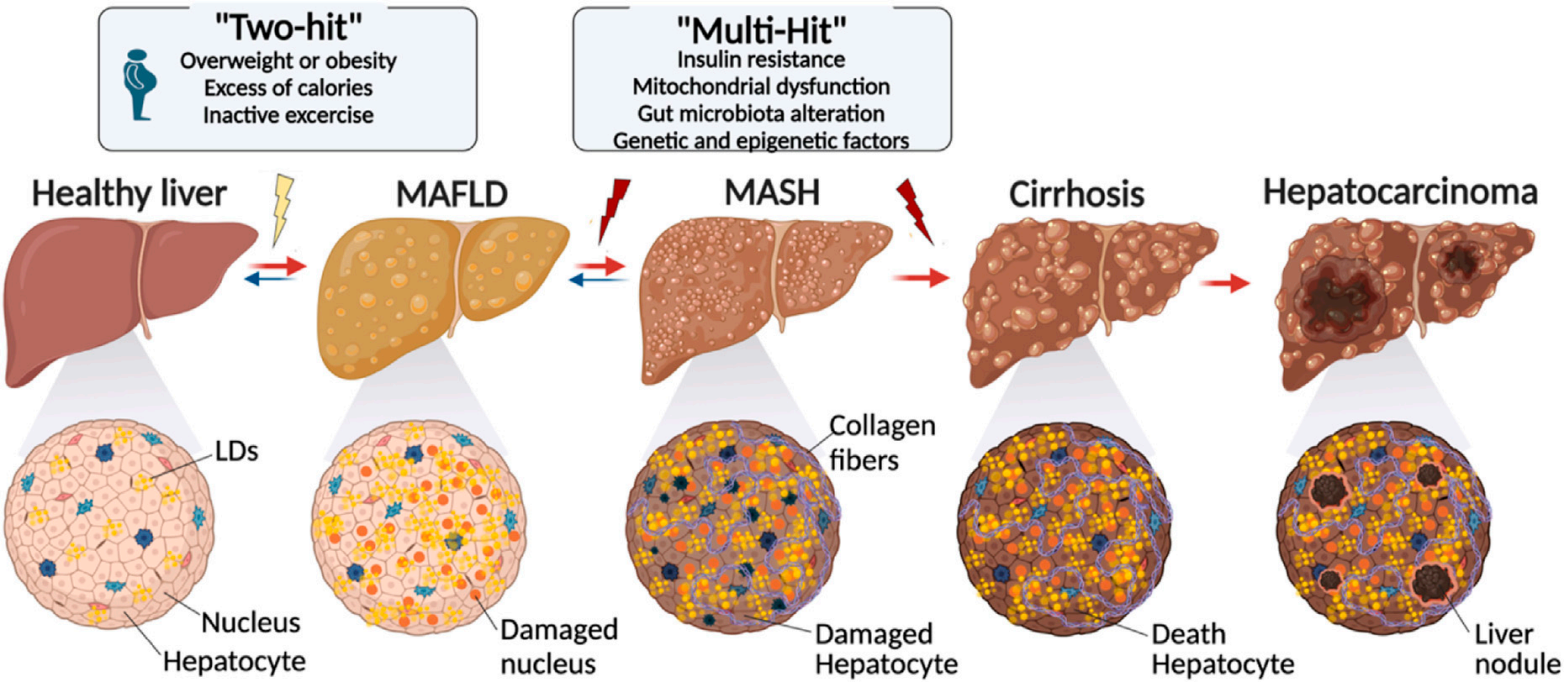
Evolution of lipid droplet over the development of liver disease. The transition from a healthy liver to advanced liver disease is characterized by a series of changes, including metabolic alterations, genetic factors, and lifestyle factors. Starting with conditions like obesity and inactivity, the proposed “Two-Hit” and “Multi-Hit” models explain the different phases leading to conditions such as MASLD and NASH, and ultimately to cirrhosis and hepatocarcinoma. Cellular damage and fibrosis process play a pivotal role in the liver disease evolution (created with BioRender.com).

While LDs are vital to liver function, an excessive amount of these cell organelles can cause metabolic dysfunction-associated steatotic liver disease (MASLD). The condition is characterized by a build-up of LDs that exceeds 5% of hepatocytes (Goh and McCullough, 2016; Wang et al., 2022), leading to interference with lipid metabolism. Excessive lipid synthesis, insufficient lipid breakdown, and abnormalities in lipid secretion are the symptoms of this disruption. With an estimated prevalence of 2%–5%, these conditions have the potential to worsen and lead to consequences such as hepatocellular carcinoma (HC) (Figure 2) (Goh and McCullough, 2016).

A variety of circumstances, including fasting, nutrition, and physical exercise, can cause an imbalance in the liver’s input and outflow of fat-soluble antioxidants, which may be the cause of the initial molecular buildup of LDs in hepatocytes (Barrows and Parks, 2006). In MASLD, hepatic lipogenesis is increased primarily as a result of insulin resistance (Smith et al., 2020) and overnutrition (Donnelly et al., 2005). The liver’s rapid accumulation of FAs may impede their conversion to TGs, leading to an increase in the production of ceramides, DAGs, and non-esterified FAs (NEFAs). These lipids can cause programmed cell death and damage liver cells (Petersen and Shulman, 2017). NEFAs, for example, can cause oxidative stress, leading to the phosphorylation of p38. This increased p38 activity enhances the expression, nuclear translocation, and transcriptional activity of p53 while decreasing those of Nrf2 thus inducing apoptosis inside the liver cells (Lei et al., 2023).

Concerning ceramides, they induce mitochondrial membrane permeability transition (MMPT), either directly or indirectly, leading to the depolarization of mitochondria. This event disrupts oxidative phosphorylation and results in the depletion of cellular ATP, ultimately culminating in cell death (Arora et al., 1997). DAGs, instead, contribute to the death of liver cells via two mechanisms: first, by activating Protein Kinase C (PKC), they disrupt cellular signaling, leading to cellular stress and inflammation, which may result to apoptosis or necrosis. Second, DAGs can play an important role in lipotoxicity by inducing ER and oxidative stress. This activates the unfolded protein response (UPR) and causes damage to cellular components, both of which can initiate cell death pathways. Overall, cell death induced by lipid overload occurs by stressing the ER and increasing ROS, which could subsequently trigger an inflammatory response (da Silva Rosa et al., 2020; Koek et al., 2011).

In addition to the mechanism afore mentioned, inefficiencies in the lipolysis process—which is crucial for the mobilization of lipids from LDs—may result in a functional impairment (Mashek, 2021). Specifically, the reduced activity of enzymes like ATGL and HSL that catalyze the TG breakdown limits the efficiency with which stored lipids may be used (Wu et al., 2011). These circumstances may accelerate the onset of metabolic dysfunction-associated steatohepatitis (MASH), a condition characterized by inflammation, fibrosis, and cellular destruction.

Other genetic variables influencing the dynamics of LD may suggest vulnerability to MASLD. Among the most important there are PNPLA3, TM6SF2, MBOAT7, SUGP1, MARC1, and GCKR.

#### 2.1.1 PNPLA3

Since 2008, numerous genome-wide association studies have linked mutations in the PNPLA3 gene to the development and severity of MASLD (Romeo et al., 2008; Anstee and Day, 2013). PNPLA3 is mostly present in the liver, particularly in hepatic stellate cells (HSCs) rather than in hepatocytes (Pirazzi et al., 2014). The rs738409 (G) variant, which produces an I148M mutation in PNPLA3, is related to increased liver fat, fibrosis (Parisinos et al., 2020), cirrhosis (Emdin et al., 2021), and higher liver enzyme levels (Kanai et al., 2018). These findings highlight the significance of understanding PNPLA3’s role in liver disease pathogenesis.

PNPLA3 in hepatocytes breaks TGs (Kumari et al., 2012), particularly those containing MUFA and PUFA, and transfers acyl groups (Huang et al., 2011). However, deleting PNPLA3 did not affect fat accumulation or lipid balance in these cells (Chen et al., 2010). On the other hand, overexpression of the PNPLA3 I148M variant resulted in increased fat accumulation since this mutation increased FA and TG synthesis while decreasing TG breakdown (Li et al., 2012). This indicates that the I148M mutation not only lowers the enzyme’s capacity to break down TG but may also provide additional functions that promote fat formation. Kumari *et al.* showed that, while PNPLA3’s ability to break down TG is limited, the I148M mutation boosts its capability to transfer acyl groups, particularly to PUFAs, resulting in increased lipid synthesis and accumulation (Kumari et al., 2012). This discovery was confirmed by a lipidomic investigation that found higher levels of PUFAs in TGs among people with the I148M mutation, indicating a role in raising MASLD risk (Luukkonen et al., 2019). Furthermore, the I148M mutation makes PNPLA3 resistant to degradation, allowing it to accumulate on liver LDs and alter fat utilization, including to the disease’s progression (Smagris et al., 2015).

Different studies have explored the possibility to target PNPLA3 as a potential therapy for MASLD, with specific attention to the I148M mutation’s gain-of-function component. In animal models, inhibiting PNPLA3 with shRNA or promoting its degradation dramatically reduced liver TG levels in mice with the I148M variant (BasuRay et al., 2019). These data indicated that PNPLA3, particularly its I148M mutation, is a suitable target for developing MASLD therapies in affected patients.

#### 2.1.2 TM6SF2

Almost 10 years ago, three different studies revealed that specific genetic variants in the transmembrane six superfamily member 2 (TM6SF2) gene are linked to MASLD (Kozlitina et al., 2014; Liu et al., 2014; Mahdessian et al., 2014). These findings highlighted TM6SF2’s role in liver cells, where its reduction leads to decreased secretion of VLDL and increased lipid storage in the liver (Kozlitina et al., 2014). Subsequently, it was confirmed that a particular TM6SF2 mutation (E167K) not only exacerbates liver fat levels and fibrosis (Daly et al., 2015) but also, paradoxically, improves insulin sensitivity (Zhou et al., 2015) and lowers serum TG levels in humans (Daly et al., 2015). This mutation results in a loss of TM6SF2 function. Moreover, experimental overexpression of TM6SF2 in mice mimicked these effects, suggesting a complex relationship between TM6SF2 activity, lipid metabolism, and liver health (Fan et al., 2016). These studies collectively point to a specific form of MASLD characterized by an unusual separation between hepatic fat accumulation and insulin response.

It was also demonstrated that patients with the E167K mutation and mice deficient in TM6SF2 exhibit a reduction in PUFAs within TGs and phosphatidylcholine (PC) in the liver, as well as in serum TG levels (Smagris et al., 2016; Luukkonen et al., 2017). Further *in vitro* studies confirmed the compromised incorporation of PUFAs in individuals carrying the E167K mutation (Smagris et al., 2016; Luukkonen et al., 2017). It was established that an increase in phospholipid saturation impedes VLDL secretion in the liver and hinders lipid absorption in the intestine (Wang et al., 2016). Consequently, the altered lipid composition stemming from TM6SF2 deficiency is likely to disrupt VLDL secretion and lipid absorption in both the liver and intestine. Thus, the pathological effects associated with a lack of TM6SF2 may stem from an imbalance in phospholipid composition (Tian and Wang, 2023).

#### 2.1.3 MBOAT7

Membrane-bound O-acyltransferase 7 (MBOAT7) has lysophospholipid acyltransferase activity and prefers PUFAs such arachidonic acid as substrates (Gijón et al., 2008). In 2015, Buch *et al.*, identified risk loci within MBOAT7 connected to alcohol-related cirrhosis, highlighting its importance in liver disease by using genome-wide association studies (GWAS) (Buch et al., 2015). MASLD cohort studies have found a substantial relationship between the RS641738T mutation in MBOAT7 and increased hepatic fat and fibrosis, which results in lower liver protein levels (Luukkonen et al., 2016a). These associations were verified by a comprehensive meta-analysis including more than a million people, which additionally linked them to decrease serum TGs and increase blood alanine aminotransferase levels (Teo et al., 2021; Caddeo et al., 2023). Moreover, MBOAT7 liver expression was found to be significantly lower in both human and mouse obesity models, implicating it in the etiology of MASLD (Helsley et al., 2019). Studies with liver specific MBOAT7 deficiency mice indicated spontaneous hepatic steatosis and enhanced fibrosis in response to dietary stressors (Luukkonen et al., 2016a). This MBOAT7 loss significantly altered hepatic lipid composition, especially reducing arachidonoyl-containing phosphatidylinositols and boosting lysophospholipids. These alterations were associated with increased *de novo* lipogenesis and fibrogenic gene expression, regardless of inflammation (Helsley et al., 2019).

#### 2.1.4 SUGP1

SUGP1 was identified in 2003 for its role in pre-mRNA splicing due to its SURP motif and G-patch domain, which suggest it can bind RNA (Fagerberg et al., 2014). Within the NCAN locus, SUGP1 and TM6SF2 are both associated with significant levels of plasma lipids, hepatic steatosis, inflammation, and fibrosis (Willer et al., 2008). Notably, the SNP rs10401969 inside SUGP1’s eighth intron is substantially associated with liver lipid levels in obese people and those with MASLD (Willer et al., 2008), albeit the exact mechanism by which this SNP regulates lipid accumulation in the liver is unknown.

According to literature, SUGP1 depletion contributed to splicing defects, especially when there is a cancer-related mutation within the SF3B1 spliceosomal gene (Zhang et al., 2019). This shows that the pathogenic consequences of SUGP1 are strongly related to alternative splicing misregulation. Kim *et al.*, revealed that the rs10401969 mutation causes SUGP1 mRNA to degrade via a process called nonsense-mediated decay. Furthermore, boosting SUGP1 levels in mice resulted in higher plasma cholesterol, whereas inhibiting SUGP1 allowed alternative splicing of 3-hydroxy-3-methylglutaryl-CoA reductase, an enzyme required for cholesterol synthesis. This effect lowered cholesterol production while increasing LDL absorption.

#### 2.1.5 MARC1

Mitochondrial amidoxime-reducing component 1 (MARC1) is an enzyme found on the outer membrane of mitochondria in mammals that includes molybdenum. It was discovered in 2008 (Gruenewald et al., 2008). This enzyme interacts with cytochrome b_5_ and its reductase to reduce N-hydroxylated compounds (Klein et al., 2012). Although its precise physiological functions are still mostly unknown, MARC1 is believed to have a role in drug metabolism and detoxification (Ott et al., 2015). The SNP called rs2642442, located in the intron of MARC1, was identified in a 2010 European population meta-analysis (Teslovich et al., 2010). This SNP is associated with lower levels of plasma cholesterol, suggesting that it may play a role in controlling lipid metabolism (Teslovich et al., 2010).

Another SNP, rs2642438, was found in more recent genome-wide association studies (Luukkonen et al., 2016b; Emdin et al., 2020). It causes a *p*. A165T variation in MARC1 and is associated with a milder form of MASLD, lower levels of aminotransferase/alanine aminotransferase, and a lower risk of cirrhosis (Emdin et al., 2020; Luukkonen et al., 2020). Although the precise mechanisms of MARC1’s activity are yet unclear, these findings suggest that it may have a protective effect against MASLD.

Elevated amounts of hepatic PC, particularly polyunsaturated ones, were found during lipidomic analysis of people bearing the *p*. A165T variation of MARC1, which may indicate a function in the remodeling of phospholipids (Luukkonen et al., 2020). This alteration is in contrast to that observed in individuals with MASLD who had TM6SF2 mutations, which similarly affect phospholipid remodeling. This suggests that the two disease processes may interact. Recent studies using MARC1-deficient mice and primary human hepatocytes showed resistance to diet-induced liver fibrosis and steatosis. Alterations in the Kennedy pathway appeared to be the mechanism by which this protective effect is mediated (Lewis et al., 2023). These findings connect phospholipids to mitochondrial activity and highlight their importance in the MASLD development. However, further investigations using mouse models are essential to fully understand MARC1’s physiological role.

#### 2.1.6 GCKR

The glucokinase regulator (GCKR), which is also known as the glucokinase regulatory protein (GKRP), is an essential component in controlling the metabolism of glucose, mainly in the liver. It regulates glucose levels by binding to glucokinase and is not affected by the conventional regulation mechanisms of substrate saturation or product inhibition (Van Schaftingen et al., 1994). A significant effect on multiple metabolic pathways was highlighted by researches, including 65 Genome-Wide Association Studies that have linked variations in the GCKR gene, specifically SNPs, with MASLD and over 130 other metabolic conditions (Anstee and Day, 2013; Goodman et al., 2020). Notably, a missense mutation known as rs1260326 is linked to reduced levels of insulin and fasting glucose but also to increased liver fibrosis, inflammation, and obesity (Anstee and Day, 2013). Because of the gene’s involvement in glycolysis, there is a paradoxical link between MASLD and glucose control. It is noteworthy that the rs1260326C variant is more prevalent in a variety of human groups, but the rs1260326T form, also known as the mutant variant rs1260326C>T P446L, is less common but has been associated with several metabolic diseases (Chen et al., 2021).

According to recent studies, depending on the individual’s diabetes status, the GKRP P446L variant impacts MASLD in two distinct ways. Individuals without diabetes who had an HbA1c < 5.7% had lower scores for MASLD activity and lobular inflammation (Kimura et al., 2022). On the other hand, those with HbA1c levels over 6.4% showed higher MASLD activity ratings and more inflammation (Kimura et al., 2022). Furthermore, in contrast to their non-diabetic counterparts who benefited from metformin therapy, diabetes individuals with the P446L mutation displayed resistance to it (Kimura et al., 2022). This suggests that among people with the GCKR mutation, there may be a complicated link between insulin resistance and fatty liver.

Together, all these genetic factors form a complex network of interactions that influence MASLD susceptibility and development. Consequently, the “two-hit” and “multiple-hit” models (Figure 2) are the two primary solutions that are put up to describe the process via which MASLD may deteriorate, even if the exact reasons for the advancement of such a condition are still not entirely understood (Buzzetti et al., 2016). According to the first, an initial build-up of hepatic lipids stimulates additional oxidative stress and inflammation, and according to the second, several variables, such as insulin resistance, mitochondrial dysfunction, changes in the gut microbiota, and genetic and epigenetic influences, simultaneously play a role in the development and course of the condition (Buzzetti et al., 2016).

In summary, excessive accumulation of LDs in the liver can trigger the development of MASLD, emphasizing the importance of maintaining a balance in lipid metabolism. The complex interplay between lipid dynamics, genetic factors, and environmental influences highlights the multifaceted nature of this liver condition, underscoring its potential to progress to severe complications such as HC.

### 2.2 LDs in metabolic dysfunction-associated steatohepatitis (MASH)

According to the American Association for the Study of Liver Diseases, fat accumulation in the liver combined with inflammation and cellular damage—possibly involving ballooning degeneration—with or without fibrosis characterizes metabolic dysfunction-associated steatohepatitis (MASH). The condition known as “ballooning” is more indicative of cellular stress than of complete cell death (Chalasani et al., 2018).

The pathophysiology of MASH is complex and includes oxidative and ER stress, dyslipidemia, changes in adipokine and cytokine production, mitochondrial dysfunction, lipotoxicity, insulin resistance, microbial translocation, and genetic predispositions. Histologically confirmed MASH is associated with an elevated risk of liver-related mortality and cirrhosis progression, particularly in cases with advanced fibrosis (Chalasani et al., 2018).

Within MASH, HSCs account for roughly 10% of the liver’s cellular population and are found in the Disse space, a microscopic area between sinusoidal capillaries and hepatocytes. They perform several essential functions, including regulating liver fibrosis and retinyl esters, which constitute vitamin A, that are stored in their cytoplasmic LDs (Wake, 1971).

In a healthy liver, this cell type is quiescent and non-proliferative; nevertheless, when the liver is damaged, it becomes active and converts from vitamin A storage cells to myofibroblasts. These myofibroblasts substantially boost the formation of extracellular matrix (ECM) due to their proliferative, contractile, inflammatory, and chemotactic properties (Puche et al., 2013; Tsuchida and Friedman, 2017). Moreover, fibrous type I and III collagens dominate the extracellular matrix (ECM), which is often interspersed with various proteoglycans, laminins, and type IV collagen (Biagini and Ballardini, 1989; Gressner, 1995; Olaso et al., 2001; Schuppan et al., 2001; Huang et al., 2017).

Approximately three-quarters of the total lipids in HSC LDs are phospholipids, retinyl ester, TGs, cholesterol, cholesterol esters, and free FAs, with retinyl ester and TGs being the most abundant (Blaner et al., 2009). A key factor in the onset of MASH is the dysregulation of lipid metabolic pathways, including lipogenesis, lipolysis, and lipophagy. While lipogenesis contributes up less than five percent of total lipid synthesis in healthy individuals, it is significantly more prevalent in MASH patients, making up to 26% of total lipid synthesis in those with this condition (Syed-Abdul, 2023).

Insulin resistance, a prevalent characteristic within MASH, promotes neutral lipolysis in adipose tissues. The excess FFAs that are produced are taken in by the liver, which stimulates the buildup of lipids (Tilg et al., 2017). Nagaya et al. reported a substantial reduction in the expression of DGAT1 and the mRNA levels of FAS and ACC1, two essential lipogenic enzymes, when comparing MASH stages to simple steatosis. High SREBP-1c levels were associated with fibrosis stages in a negative correlation. This suggests that ‘burned-out’ MASH and SREBP-1c downregulation are linked to reduced hepatic steatosis in advanced MASH and its target genes (Nagaya et al., 2010). Additionally, it was hypothesized that cholesterol buildup may exacerbate cellular dysfunction (Yamaguchi et al., 2007; Wouters et al., 2010). When cholesterol is esterified, it may be preserved quite safely. However, even if its quantity only slightly increases, the presence of free, non-esterified cholesterol in the liver may seriously damage many different kinds of cellular functions and organelle structures (Gan et al., 2014). The specific mechanisms through which cholesterol can cause lipotoxicity and contribute to the development of MASH are not yet well understood (Ioannou, 2016). However, in contrast to what has been observed in people or mice with simple steatosis, new researches are demonstrating the production of cholesterol crystals within LDs of steatotic hepatocytes in individuals with MASH as well as in a MASH mice fed with a high-fat diet (Ioannou et al., 2013). Furthermore, it has been noted that swollen Kupffer cells position themselves alongside dead steatotic hepatocytes that have cholesterol crystals within them. This action should be necessary to break down the remaining LDs in these hepatocytes and create “crown-like structures” that resemble visceral adipose tissue that becomes inflamed (Strissel et al., 2007). Recent research has also demonstrated that cholesterol crystals activate the NLRP3 inflammasome, suggesting a mechanism by which the exposure of Kupffer cells to cholesterol crystals could lead to chronic inflammation and MASH (Rajamäki et al., 2010).

HSCs can be also triggered by an excess of free cholesterol by activating signaling pathways that lead to fibrogenesis and inflammation (Arguello et al., 2015). FFAs, ROS, and other lipid metabolic byproducts affect this cell population activation (Wang et al., 2020). Plin5 may inhibit HSC activation, according to studies (Lin and Chen, 2016), although little is known about how PLIN5 affects active HSC apoptosis. It is interesting to note that, as demonstrated by Yin et al., PLIN5 overexpression in HSCs significantly accelerates LD formation compared to cells with aberrant PLIN5 expression. PLIN5 triggers the caspase and AMPK cascades, which cause HSCs to die and stop growing, respectively. Therefore, all of these data point to the idea that HSC lipogenesis might be restored with the aim of lowering HSC activity and reversing fibrogenesis (Tsukamoto et al., 2008).

In summary, MASH is characterized by an excessive LD accumulation within liver cells, which is triggered by an unbalanced lipid metabolism and storage system. This buildup is a characteristic of the disease, causing inflammation, liver cell damage, and even fibrosis. In MASH, LDs are more than passive fat deposits; they actively contribute to the disease’s cellular stress and inflammation. Their presence is directly connected with the development of basic steatosis to more severe liver damage in MASH, highlighting their importance in the disease’s pathogenesis.

### 2.3 LDs in liver cirrhosis

Cirrhosis is a chronic liver condition defined by the gradual replacement of healthy liver tissue with fibrotic scar tissue, according to scientific recommendations (Anthony et al., 1978; Ferrell, 2000; Wanless et al., 2000; Elsharkawy et al., 2005). This change is the result of long-term liver damage, which is frequently brought on by autoimmune diseases, alcohol abuse, viral hepatitis, MAFLD, and other chronic illnesses (Deutsch et al., 2013; Selmi et al., 2011; van et al., 2007; Poupon et al., 2006). Despite the diverse origin of liver cirrhosis, there are common pathological features across all cases, including hepatocyte degeneration and necrosis, the replacement of normal liver tissue with fibrotic tissue, and the development of regenerative nodules, leading to reduced liver functionality (Anthony et al., 1978; Ferrell, 2000; Wanless et al., 2000; Elsharkawy et al., 2005).

Liver chronic inflammation is critical because it triggers the activation of macrophages and myofibroblasts within the same tissue, which leads to the accumulation of collagen and the advancement of fibrosis. This changes the liver’s normal architecture, cutting off the flow of blood, inducing fibrotic nodules to develop, and destroying the connection between hepatocytes and sinusoids. Long-term damage also causes the loss of hepatocytes and reduces the liver’s metabolic activity (Northup et al., 2021; Whitfield et al., 2022).

Within this pathological condition, lipids and LDs play a significant role in the development of cirrhosis. They serve as signaling molecules that control immune responses and promote inter-organ communication in addition to being necessary for cellular architecture and energy sources (Phillips et al., 2009). Moreover, certain lipid byproducts act as inflammatory mediators and lipids’ interactions with Toll-like receptors were shown to trigger inflammation (Köberlin et al., 2015). FA amounts are elevated, which is consistent with decompensated cirrhosis (Moreau et al., 2020). The progression from simple hepatic steatosis to cirrhosis is marked by a series of significant cellular transformations, including hepatocyte swelling, ER expansion, cytoskeletal damage with the appearance of Mallory-Denk bodies, parenchymal tissue damage, and LD accumulation (Argo et al., 2016). The latter results from malfunctioning LD production and degradation processes, which raise the likelihood of developing cirrhosis by encouraging steatosis, inflammation, and fibrosis in addition to increasing lipid buildup within cells (Mashek, 2021). The liver experiences an architectural alteration in this disorder, leading to an increase in hepatic stiffness (Tatsumi et al., 2015). Hepatocyte nuclei may become distorted as a result of LD amount, which could result in internal mechanical stress. According to recent research, there appears to be a relationship between hepatocyte functioning and nuclear deformation, suggesting that LDs are intracellular sources of mechanical stress (Guixé-Muntet et al., 2020). Because the nucleus serves as a major point to integrate mechanical stress across many cell types, nuclear deformation brought on by intracellular alterations may facilitate the development of cirrhosis (Heo et al., 2023). In general, stiff intracellular inclusions may constitute mechanical stressors, indicating that nuclear deformation plays a role in the development of a cirrhotic condition.

Concluding, due to their complex role, LDs are both a hallmark of the liver’s battle with chronic damage and an essential part of its defense systems. Cirrhosis is the result of long-term liver injury, characterized by scarring and reduced function. LDs are involved in the disease but may also provide protective benefits. Their buildup can signal and worsen metabolic disruptions, adding to the stress and inflammation that drive fibrosis. Nonetheless, by sequestering harmful lipids, LDs may reduce lipotoxicity, providing a type of cellular defense. This dual role highlights the complexity of LDs in liver cirrhosis, emphasizing their relevance in both the development and potential prevention of liver damage.

### 2.4 LDs in hepatocellular carcinoma

**FIGURE 3:**
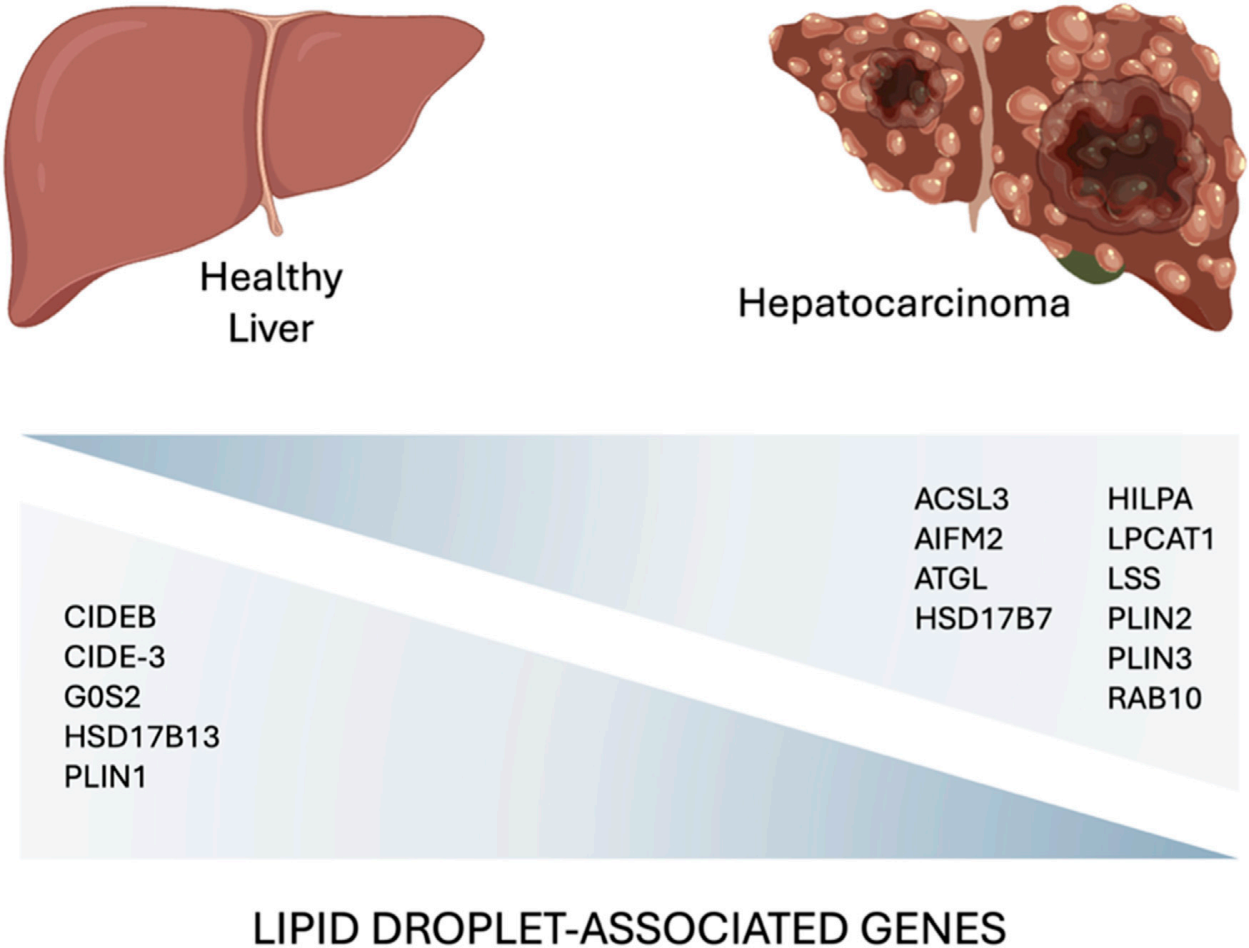
A comparative diagram highlighting key lipid droplet-associated genes involved in the hepatocarcinoma development (Min et al., 2011; Shyu et al., 2018; Povero et al., 2023; Zhang et al., 2023) (created with BioRender.com).

Accumulation of LDs has been associated with higher tumor aggressiveness, drug resistance, and poor prognosis in many cancers (Tirinato et al., 2015; Tirinato et al., 2017; Cruz et al., 2020; Tirinato et al., 2021). Lipid metabolism is often severely impaired in tumor cells that exhibit increased *de novo* lipogenesis, fatty acid uptake, and fatty acid oxidation. These processes lead to high intracellular lipid levels that allow tumor cells to synthesize plasma membranes, to produce ATP, and to mitigate oxidative stress as lipid composition and saturation can influence ROS tolerance thus enhancing cancer cell survival (Cheng et al., 2022). Since the liver is the core organ of lipid metabolism, it is not surprising that LDs play an important role in both the development and progression of liver cancer. In particular, changes in the expression level of LD-associated proteins have been correlated with LD metabolism in liver cancer. Data from public databases, revealed transcriptional change in tumor and normal liver tissues, and found that 15 LD-associated proteins may involve in the progression of HCC (Figure 3).

The same gene signature was also found to be modulated in mouse fatty liver and human MASLD liver samples suggesting the involvement of LD-associated proteins in MASLD-HCC progression (Zhang et al., 2023).

While hypoxia inducible lipid droplet-associated (HILPDA) is strongly connected with poor mortality in patients with MASH-driven HC, it has also been observed to support the formation of 3D epithelial spheroids and the viability of human hepatoma cells under anoxic culture conditions. Furthermore, hypoxic lipid peroxidation and apoptosis were enhanced by HILPDA deficiency, which drove PUFAs to membrane phospholipids and saturated FAs to ceramide production (Povero et al., 2023).

Additionally, it was discovered that DGAT1 protein levels were up in liver cells that overexpressed HILPDA, and this was accompanied by an increase in fat storage (de la Rosa Rodriguez et al., 2021). DGAT1 is important for the maintenance of HC *in vitro* and its silencing regresses HC cells to a stem cell-like phenotype (Chang et al., 2016). More recent data suggests that ATGL may have a special tumor suppressive function. As a result, in both a HC mouse and human models, ATGL is downregulated. Using its acetylation mediated by the PPAR-α/p300 axis, Di Leo *et al.* discovered that ATGL stabilized the tumor-suppressor p53 in HC cells. This axis is also known to drive the metabolic process (Di Leo et al., 2019). This is significant since it is known that p53 affects lipid metabolism, which is a non-canonical function.

Cell death-inducing DFF45-like effector-3 (CIDE-3) is another target that has been shown to be downregulated in HC. According to studies conducted by Min *et al.*, there was a positive correlation between the expression of CIDE-3 and HC differentiation, and there was a decrease in CIDE-3 expression in HC tissue when compared to nearby normal tissues (Min et al., 2011). Specifically, lower CIDE-3 expression levels were associated with poorly differentiated HC tissue. In contrast, in *vitro* study overexpressed CIDE-3 inhibited proliferation and induced apoptosis in HC cells (Min et al., 2011).

As was previously mentioned, one of LDs’ primary functions is to provide protection against oxidative stress. ROS primarily interacts with conjugated double bonds of PUFAs, leading to lipid peroxidation that, in certain cases, affects normal cell function and results in cell death (Jomova et al., 2023). Because LDs are less susceptible to peroxidation than cellular membranes, phospholipases can liberate PUFAs from membranes under oxidative stress situations. When the amount of PUFAs exceeds the capacity of LDs, ferroptosis develops within cancer cells (Dierge et al., 2021). This could explain the observation of decreased serum levels of PUFAs in the blood of patients with HC (Vlock et al., 2020; Lewinska et al., 2021).

In conclusion, LDs accumulation correlates with increased tumor aggressiveness and drug resistance, underscoring the critical importance of lipid metabolism.

## 3 Conclusion

In conclusion, this review is a tentative to highlight the role of LDs in the progression of hepatic diseases, from MASLD to HC. The intricate relationship between LDs and liver health is underscored by their dual role in both supporting cellular energy needs and contributing to disease pathogenesis when dysregulated. The accumulation of LDs, while essential for energy storage and metabolic regulation under normal conditions, can lead to detrimental outcomes in the context of hepatic diseases. This is particularly evident in the progression from MASLD to MASH, liver cirrhosis, and ultimately HC, where altered lipid metabolism, oxidative stress, and inflammation play pivotal roles.

The involvement of LD-associated proteins in the development and progression of HC further emphasizes the importance of lipid metabolism in liver cancer. Understanding the biological LD functions and their regulatory mechanisms offers potential therapeutic targets for mitigating the progression of liver diseases and improving patient outcomes.
